# Supplementary material for: Antenatal care component utilization and associated factors among pregnant women in Ethiopia: Multilevel analysis of Ethiopian Mini Demographic and Health survey 2019
Source: PLoS One. 2024 May 29;19(5):e0303118. doi: 10.1371/journal.pone.0303118 (PMC11135780; doi:10.1371/journal.pone.0303118)
Supplement: Attachment — Submitted filename: Comment.docx [file pone.0303118.s001.docx]

The manuscript lacks novelty; a study conducted using the same data uncover the aim of this study (https://doi.org/10.1371/journal.pgph.0000584 ).
