# Supplementary material for: Antenatal care component utilization and associated factors among pregnant women in Ethiopia: Multilevel analysis of Ethiopian Mini Demographic and Health survey 2019
Source: PLoS One. 2024 May 29;19(5):e0303118. doi: 10.1371/journal.pone.0303118 (PMC11135780; doi:10.1371/journal.pone.0303118)
Supplement: Attachment — Submitted filename: Rebuttal Letter.docx [file pone.0303118.s002.docx]

# **Rebuttal Letter**

We are grateful that you have considered our appeal request and willing to reconsider the manuscript.

**Requested points from your office:** “Please accompany this resubmission with a detailed rebuttal letter that includes a point-by-point response to the concerns raised by the Academic Editor and details on the revisions carried out on the manuscript since its original submission”.

**The concern raised by the academic editor**: “The manuscript lacks novelty; a study conducted using the same data uncover the aim of this study (<https://doi.org/10.1371/journal.pgph.0000584> )”.

The only concern raised from the academic editor is that another study, <https://doi.org/10.1371/journal.pgph.0000584>, from the same data uncovered the aim of the current study.

We have clearly put the difference of both studies below point-by-point.

**Our current work**

1. **Title of our current work**: Antenatal Care Component Utilization and Associated Factors among Pregnant women in Ethiopia: Multi level Analysis of Ethiopian Mini Demographic and Health survey 2019
2. **Objective:** The objective of this study was to identify the proportion of pregnant women who received adequate Antenatal care (ANC) components and associated factors among Ethiopian women.

- In this case we identified what proportions of pregnant women, who have history of at least one Antenatal care visit, have received adequate components/contents of the care. To judge the adequacy of the received components of ANC we asked whether the woman has got history of measured blood pressure, taking blood sample, taking urine sample and given/bought iron tablets/syrup during the pregnancy.

3. **The outcome** variable in this study was receipt of the four components of ANC among women with at least one ANC visit during the last pregnancy and dichotomized in to “Yes” if the woman received the four components and “No” if she missed at least one of the components.

**The previously published work**

# 1. **Title of the previously published article**: Late antenatal care utilization in Ethiopia: The effect of socio-economic inequities and regional disparities

2. **Objective** of the previously published article: aimed to investigate utilization of ANC the effect of socio-economic inequities and regional disparities in Ethiopia.

Here what they have explicitly studied was the proportion pregnant women who received ANC (any number of visit) during their last pregnancy. In addition to this, they have also identified the proportion of late ANC initiation (first ANC visit later than 4 months of pregnancy).

- Both of their objectives are completely different from our current study

3. **Outcome variable:** A binary outcome variable was the first ANC booking/initiation to the health facility within five years of the survey which was coded as “0” if early and “1” if late. ANC booking was taken as early if initiated before 4 months and late if then after.

Therefore, from the above listed points it is clear that both studies are different and there is no single point that questions novelty of our current study compared with the mentioned article.

Thank you
