# Supplementary material for: Antenatal care component utilization and associated factors among pregnant women in Ethiopia: Multilevel analysis of Ethiopian Mini Demographic and Health survey 2019
Source: PLoS One. 2024 May 29;19(5):e0303118. doi: 10.1371/journal.pone.0303118 (PMC11135780; doi:10.1371/journal.pone.0303118)
Supplement: Attachment — Submitted filename: First.docx [file pone.0303118.s003.docx]

First, we would like to say thanks for editor who gave this chance to revise the manuscript entitled “Antenatal Care Components Utilization and Associated Factors among Pregnant women in Ethiopia: Multi level Analysis of Ethiopian Mini Demographic and Health survey 2019.” Next to this, i would like to proceed to provide few comments and suggestions for authors as follows.

**On methods**

1. In line 120 …Independent variables… please, classify Independent variables in to two subgroups as follow

**Individual level Variables:**

**Community level variables:**

2. In line 120 …Independent variables… if possible, you will add “sex of household head and religion” as predictor’s or Independent variables.

**On results**

3. In line 206 ….Factors associated with receiving ANC contents… you were employed multilevel logistic regression model. In multilevel logistic regression model, you were doing random effect and model comparison in order to select the best fitted model. Please state the results of each model in the form of the following templates. These helps readers to access the message easily and clearly.

| Parameters | model I | model II | model III | Model IV |
| --- | --- | --- | --- | --- |
| ICC (%) |  |  |  |  |
| Model fitness |  |  |  |  |
| **AIC** |  |  |  |  |

4. What are the criteria to select the best fitted model in multilevel logistic regression model? (i.e. Model I, model II, model III and Model IV)

**On discussion**

5. Your discussion was clearly written, precise and well stated. But, in order to make your discussions strong and scientifically sounded, as much as possible try to cites some scientific literatures for your justifications or explanation throughout your discussion.
6. On line 301-303 …..”This might be because of those in better wealth status have access to better services and more confident to ask for their right”…… you were stating the above justification. Since, most ANC packages were free of charges or fees. Does the stated explanation was enough? There were several literatures that support this finding, please revise those literatures and add more justification.
