# Supplementary material for: Antenatal care component utilization and associated factors among pregnant women in Ethiopia: Multilevel analysis of Ethiopian Mini Demographic and Health survey 2019
Source: PLoS One. 2024 May 29;19(5):e0303118. doi: 10.1371/journal.pone.0303118 (PMC11135780; doi:10.1371/journal.pone.0303118)
Supplement: Attachment — Submitted filename: Response to reviewers1.docx [file pone.0303118.s004.docx]

**Point by point response to the reviewers’ comments**

First of all, we would like to say thank you to all who have participated in reviewing our manuscript and your comments are addressed as follows.

1. Please ensure that your manuscript meets PLOS ONE&#39;s style requirements, including those for file naming. The PLOS ONE style templates can be found at https://journals.plos.org/plosone/s/file?id=wjVg/PLOSOne_formatting_sample_main_body.pdf and https://journals.plos.org/plosone/s/file?id=ba62/PLOSOne_formatting_sample_title_authors_affiliations.pdf

**Response:** Thank you for your advice and the manuscript is prepared per PLOS ONE’s style requirement.
2. Please upload a copy of Figures 1 and 2 which you refer to in your text on pages 19 and 20. Or if the figure is no longer to be included as parts of the submission please remove all reference to it within the text.

**Response:** thank you, the figures are uploaded

Reviewer #1: &bull; there is grammar and spelling error throughout the document. Thus, the language editing need to be carefully correct errors in grammar, punctuation, and consistency
Introduction

**Response:** Thank you for you valuable comments and we have edited the manuscript accordingly.
&bull; The authors would do better to disclose the evidence gap in the earlier research and addressed by the current study in the introduction section.

**Response:** thank you for the comment and the introduction part is revised accordingly.
&bull; There are few sentences about adequate ANC in the introduction section. Better to introduce the concept of adequate ANC components and its contribution to maternal and child health in the introduction section.

**Response:** Thank you for this important comment and we have added more about ANC components in the introduction.
Model fitness:
&bull; The authors provided no information regarding whether or not the data was fitted to the model. Therefore, it will be beneficial to mention that the goodness of fit test taken into account and to state whether the data was fitted to a multi-level logistic regression model.

**Response:** Thank you very much for this valuable comment. We incorporated a separate table regarding model fitness in the manuscript and we hope that it addresses the issue.
Discussion
&bull; The novel idea in this study the adequate ANC components was not well discussed in terms of its implication for health professional, program planners, and policy makers. Others pertinent findings of the study need to be interpreted and discussed for their program and policy implication at different level.

**Response:** Thank you for this brilliant comment and we added further elaboration and discussion regarding the ANC components adequacy.
Limitation
&bull; The authors did not go into much detail about the limitation of the study. For instance, rather than client-related considerations, contextual factors such as the capacity of healthcare facilities and the competency of healthcare professionals have a greater impact on the adequate of ANC components. Thus, these and other study limitations must be addressed.

**Response:** Thank you for your comments and suggestions. We have incorporated the points raised and they are really important.

Reviewer #2: First, we would like to say thanks for editor who gave this chance to revise the manuscript entitled &ldquo;Antenatal Care Components Utilization and Associated Factors among Pregnant women in Ethiopia: Multi level Analysis of Ethiopian Mini Demographic and Health survey 2019.&rdquo; Next to this, i would like to proceed to provide few comments and suggestions for authors as follows.
On methods
1. In line 120 &hellip;Independent variables&hellip; please, classify Independent variables in to two subgroups as follow
Individual level Variables:
Community level variables:

**Response:** thank you very much for your valuable comments and suggestion. The independent variables are separately discussed per their level.
2. In line 120 &hellip;Independent variables&hellip; if possible, you will add &ldquo;sex of household head and religion&rdquo; as predictor&rsquo;s or Independent variables.

**Response:** Thank you for this golden idea and comments. We rerun the model by including sex of the head of household and religion of the participants. Accordingly, we found them significant and we reported the estimates of the other independent variables considering the inclusion of the two variables.
On results
3. In line 206 &hellip;.Factors associated with receiving ANC contents&hellip; you were employed multilevel logistic regression model. In multilevel logistic regression model, you were doing random effect and model comparison in order to select the best fitted model. Please state the results of each model in the form of the following templates. These helps readers to access the message easily and clearly.
Parameters model I model II model III Model IV
ICC (%)
Model fitness
AIC
**Response:** we really appreciate your suggestions and comments. Per your suggestion we separately included the model diagnosis parameters as table three.
4. What are the criteria to select the best fitted model in multilevel logistic regression model? (i.e. Model I, model II, model III and Model IV)
&emsp;

**Response:** we selected the variable with the smallest AIC value and the final model was the best fitted model.
On discussion
5. Your discussion was clearly written, precise and well stated. But, in order to make your discussions strong and scientifically sounded, as much as possible try to cites some scientific literatures for your justifications or explanation throughout your discussion.

**Response:** thank you for your suggestion and comment; we have modified accordingly.
6. On line 301-303 &hellip;..&rdquo;This might be because of those in better wealth status have access to better services and more confident to ask for their right&rdquo;&hellip;&hellip; you were stating the above justification. Since, most ANC packages were free of charges or fees. Does the stated explanation was enough? There were several literatures that support this finding, please revise those literatures and add more justification.

**Response:** Thank you for your deep insight and suggestions. We have corrected it accordingly

Reviewer #3: Title: Antenatal Care Components Utilization and Associated Factors among Pregnant Women in Ethiopia: Multi level Analysis of Ethiopian Mini Demographic and Health survey 2019
General comments
What does Antenatal Care Components Utilization mean? And how that is different from a lot of evidence already published from the same dataset of MEDHS 2019
It looks like this study is adding another duplication of already over-duplicated information and I don&rsquo;t see any reason to have it again. This is the most researched topic.

**Response:** Thank you for your concern and comment. We have reviewed if there is a similar study from the same dataset and found no similar study with objective. Rather, other articles addressed the area diffrently
Abstract
The abstract introduction is meaningless: not mentioning why to conduct the study, no gap, no something this study going to do, and fake objective stating &lsquo;adequate antenatal care&rsquo;. I recommend rewrite

**Response:** thank you for your comment and we rewrote the introduction part of the article by incorporating additional points
The method section should include information on multilevel model (what, why&hellip;etc)
The ANC of 55% is very from real figure from other studies which is around 48% and the pooled prevalence of 63.77%?

**Response:** Thank you for your comment. The reason it is 55% in this study is that we have considered only four of the components namely blood pressure measure, blood and urine sample taking and iron supplementation.
Positive and negative associations not separated?

**Response:** Thank you for your comment and the point is addressed accordingly.
The abstract conclusion do not make any sense whatsoever, redraft with right summary, practical recommendation and limitation.

**Response:** thank you for your suggestion and it is addressed accordingly.
Introduction
- The introduction could be clearer and more concise. Consider restructuring the section.
- Use academic writing style. Your document is full of non-academic terms e.g. &lsquo;sluggish decline&rsquo; on paragraph 2

**Response:** thank you for the comment and it is addressed
- It provides extensive background information about ANC without discussing and comparing the magnitude of the problem and making logical arrangements on the severity categories.
- It might be more effective if the text is more concise and focused on the most crucial information, including clearly identifying the gap in knowledge that this study is addressing. Many studies already discussed this information and it is already overwhelming. Thus, you need specific importance to continue with study.

**Response:** Thank you for the comments and suggestions. The points are addressed accordingly.
- There is a lack of structure in presenting previous studies and findings. Organizing these findings more systematically would allow for a clearer understanding of the previous work in this area and the contribution of the current study.

**Response:** thank you for the comment and we have addressed them
- The final paragraph should more succinctly summarize the rationale for the study and the specific objectives. The current text is extensive and could be more concise and focused.
- It would be better to arrange the section with what was presented to be ANC components
- Or include enough details about each essential component you are trying to talk about.

**Response:** thank you for your valuable comments and insights and the points are addressed.
Methods
- The method has enough details but is still highly confusing. It doesn&rsquo;t contain information on ANC components. Overall, it is really difficult to understand this study and know what the authors trying to do.

**Response:** thank you and we addressed the comments
- Re-write your methods based on components that we expect in the result section

**Response:** thank you and the comments are incorporated.
- There was no detailed structure presented regarding the statistical methods
- Detailed definition of each variable not included
- Include an explanation regarding the possibility of bias and multicollinearity need to be addressed

**Response:** thank you for your insights and comments. We have addressed your comments
-
Results
- The result presentation is supposed to follow the components indicated under the method section. Please, correct the method section and results accordingly.

**Response:** Thank you for the suggestion and we have modified it accordingly.
- The interpretation of the analysis results needs to be clearer, highlighting the significance of the findings

**Response:** thank you for your comment and we have addressed it
- Provide a clearer explanation of the statistical results, ensuring that terms and statistics are understandable for readers without a statistical background.

**Response**: thank you for your valuable comments and we have modified the specific area accordingly
- Ensure consistency in the presentation of results, using a uniform format for presenting statistical data and other information.

**Response**: thank you and we have improved it accordingly
- At the end of the results section, provide a concise summary of the main findings and their significance.
**Response:** thank you for your time and valuable comments. We have included it accordingly
Discussion
- include in-depth comparison with existing literature, especially contrasting findings and methodologies. Further, discussing how this study adds to the existing body of knowledge could be more explicit. Please, focus because this information is missing, while it is the base for understanding your study.

**Response:** Thank you for the comments and we have addressed it
- Include all limitations related methods and data such as methodology used, data, sampling, representatively, inclusion criteria, or analysis methods

**Response**: thank you for your valuable comments and it is well addressed
Conclusion
- The main conclusions of the study should be presented more clearly and concisely. It&#39;s essential to summarize the key findings and their implications straightforwardly.
- The conclusion should include more detailed recommendations for future research.
- The conclusion could elaborate more on the policy implications of the findings. How should policymakers approach

**Response:** Thank you for your golden comments and we have incorporated them accordingly.
