# Supplementary material for: Antenatal care component utilization and associated factors among pregnant women in Ethiopia: Multilevel analysis of Ethiopian Mini Demographic and Health survey 2019
Source: PLoS One. 2024 May 29;19(5):e0303118. doi: 10.1371/journal.pone.0303118 (PMC11135780; doi:10.1371/journal.pone.0303118)
Supplement: Attachment — Submitted filename: Response to reviewers.docx [file pone.0303118.s006.docx]

**Point by point response to the academic editor’s comments**

First of all, I would like to say thank you for your essential comments and your comments are addressed as follows.

1. Comments 1 and 2: What is this number?

**Response**: thank you for your critical observation and valuable comments. These numbers where the level of association I calculated during the previous version. Later on after the reviewers comment, I included religion and sex of the head of the household in multivariable analysis after which the value of AOR was changed. These numbers where remained from the former version by my own error and now I have corrected it. Thank you

2. Comment 3: Your write up here is not understandable. Please revise

**Response:** Thank you for your constructive comment and now I have revised it.

3. Comment 4: Yes that is well known. So how it should be improved? Any possible recommendation from the implication of your study?

**Response:** thank you for your comment and I have included possible recommendations based on the findings

4. comment 5: Write in standard form

**Response:** thank you for your comment and it is corrected now.

Comment 6: What is the difference between this statement and the previous one?

**Response:** Thank you for your meticulous comment and corrected by removing the duplicated statement.

5. Comment 7: For which visit was the outcome measured? Not clear

**Response:** Thank you for your comment. The outcome of interest including the four components was considered irrespective of the order or number of the visit. It can be at any of the visits.

6. Comment 8: Please group the figure to connect the boxes together and submit it as a supplementary file.

**Response**: Thank you for your comment and I have submitted as supplementary file per your comment.

7. Comment 9: But you operationalized as Yes/No, see above. When you say it is adequate?

**Response:** when it is adequate it is “Yes” and when it is in adequate it is “	No”

8. Comment 10: Please use “≥” or “≤”for accordingly

**Response**: thank you for your comment and it is corrected accordingly.

9. Comment 11: The way you interpret the results is confusing and not understandable. Please revise it

**Response:** Thank you for your comment and it is revised now.

10. Comment 12: Any potential competing interests, Funding information, Author contributions

**Response:** Thank you for your comment and these components are included in the manuscript
